# Prevalence of methicillin-resistant *Staphylococcus aureus* in healthy Chinese population: A system review and meta-analysis

**DOI:** 10.1371/journal.pone.0223599

**Published:** 2019-10-24

**Authors:** Man Wu, Xiang Tong, Sitong Liu, Dongguang Wang, Lei Wang, Hong Fan

**Affiliations:** Department of Respiratory and Critical Care Medicine, West China Hospital/West China School of Medicine, Sichuan University, Chengdu, China; Rabin Medical Center, Beilinson Hospital, ISRAEL

## Abstract

**Objective:**

To comprehensively determine the prevalence of MRSA in healthy Chinese population, the influencing factors of MRSA colonization and its antibiotic resistance.

**Methods:**

Articles that studied prevalence or influencing factors of MRSA carriage in healthy Chinese population were retrieved from PubMed, Ovid database, three Chinese electronic databases. The pooled prevalence of MRSA, its antibiotic resistance and influencing factors were analyzed by STATA12.0.

**Results:**

37 studies were included. The pooled prevalence of MRSA was 21.2% (95% CI: 18.5%-23.9%), and the prevalence of S.aureus was 15% (95% CI: 10%-19%), with a significant heterogeneity (MRSA: I^2^ = 97.6%, P<0.001; S.*aureus*: I^2^ = 98.4%, P < 0.001). In subgroup analysis, the pooled prevalence of MRSA was 28% (95%CI: 10%-51%) for Livestock-related workers, 18% (95%CI: 11%-26%) for children, 20% (95%CI: 12%-29%) for healthcare workers, 7% (95%CI: 3%-13%) for community residents. The prevalence of MRSA in studies with oxacillin disk diffusion method (28%, 95%CI: 21%-35%) seemed higher than that with the mecA gene method(12%, 95%CI: 7%-19%). MRSA in studies conducted in Taiwan was more common than in Mainland China and Hong Kong. Similar results were found in meta-regression. Influencing factors for MRSA colonization were noted in seven eligible studies, they included younger age (OR: 3.54, 95% CI: 2.38–5.26; OR: 2.24, 95% CI: 1.73–2.9), attending day care centers (DCCs) (OR: 1.95, 95% CI: 1.4–2.72; OR: 1.53, 95% CI: 1.2–1.95), flu vaccination (OR:1.73, 95% CI: 1.28–2.35), using antibiotics within the past year (OR: 2.05, 95% CI:1.35–3.11), residing in northern Taiwan (OR: 1.45, 95% CI: 1.19–1.77), regular visits to health care facility (OR: 23.83, 95% CI: 2.72–209.01), household member working in health care facility (OR: 8.98, 95% CI:1.4–55.63), and contact with livestock (OR: 6.31, 95% CI: 3.44–11.57). Moreover, MRSA was found to be highly resistant to penicillin, ampicillin, erythromycin, and clindamycin, with a pooled resistance ratio of 100, 93, 88, and 75%, respectively. However, no resistance were noted to vancomycin.

**Conclusion:**

The pooled prevalence of MRSA was considerably high in health Chinese population. Additionally, these strains showed extreme resistance to penicillin, ampicillin, erythromycin and clindamycin. Public MRSA protection measures and the surveillance of MRSA should be strengthened to reduce the spread of MRSA among hospitals, communities, and livestock.

## Background

*Staphylococcus aureus* (S.*aureus*) is one of the main causes of hospital and community-acquired infections, resulting in serious consequences, and the disease ranges from skin infections to Septic shock[1]. Following the introduction of penicillin in 1940, S.*aureus* resistance appeared, leading to the development of semisynthetic penicillins such as methicillin. In 1960, methicillin-resistant *Staphylococcus aureus* (MRSA) was clinically identified. Poor infection control measures and continued indiscriminate exposure to antibiotics in humans and animals lead to MRSA transmission[2]. In recent years, the prevalence of MRSA is rising. The infection due to the MRSA strains has a higher mortality rate than the infection caused by the methicillin-sensitive *Staphylococcus aureus* (MSSA) strains, which brings great difficulty to treatment[3, 4].

MRSA acquires methicillin resistance by expressing a penicillin-binding protein (PBP2a) with reduced affinity for most available beta-lactam agents, including methicillin, which is encoded by mecA gene located in a mobile genomic element known as the staphylococcal cassette chromosome mec (SCC*mec*). New drug resistance genes have been discovered in recent years (*mecB*, *mecC*, and/or *mecD*)[2]. The MRSA colonization and infection has appeared from hospitals to the community and further to animals, so MRSA is no longer only anosocomial pathogen. Depending on the genotype, MRSA can be divided into community-acquired MRSA (CA-MRSA) and hospital-acquired MRSA (HA-MRSA), CA-MRSA strains are commonly sensitive to a variety of non-beta-lactam antibiotics and usually carry SCC*mec* type IV (less common, type V) and Panton-Valentine leukocidin (PVL) gene. While HA-MRSA strains are resistant to a variety of antibiotics, and are most associated with type I, II and III SCC*mec*[5, 6].

S.*aureus* colonization is a global phenomenon affected by various factors, not limited to age, health, economic status and country. S.*aureus* may be colonized in multiple body parts, but the anterior nares are the most stable colonization site. S.*aureus* colonization has been identified as an important risk factor for the development of S.*aureus* infection in community and hospital settings[7, 8]. In the past few years, the colonization rate of MRSA in healthy hosts increased significantly and may play an important role in the spread of MRSA in community and hospital settings[9]. Previous studies have shown that the demographic (e.g. age, gender, region), environmental (e.g. crowded or medical environments, animal contact), and host factors (e.g. immunity, received antibiotics) may be influence factors for MRSA carriage[7, 10]. Therefore, it is important to understand the prevalence of MRSA in healthy population at the country level to support effective prevention and control strategies.

In recent years, extensive investigative researches were performed in China on the prevalence of MRSA in healthy people, but the results are quite different with limited sample sizes. Therefore, it is necessary to conduct a systematic review and meta-analysis to comprehensively determine the prevalence of MRSA in healthy Chinese population, the influencing factors of MRSA colonization and its antibiotic resistance, which may help to establish public health interventions to reduce MRSA infection.

## Materials and methods

### Inclusion criteria

The following are the inclusion criteria in our meta-analysis: (1) the subjects were healthy Chinese population (Eligible participants with no acute medical problem); (2) observational studies including cross-sectional, prospective, and retrospective study (**e.g.** cohort and case-control studies); (3) provided total number of S.*aureus* and MRSA strains, and the total sample size; (4) Nasal or nasopharyngeal specimens. The following exclusion criteria were applied: (1) the study objects were special population (pregnant women, residents of nursing homes, infants); (2) previous studies were repeated; (3) editorial articles, meta-analyses, abstracts, letters or reviews; and (4) reported outbreak epidemiological data.

### Search strategy

This meta-analysis followed PRISMA (Preferred Reporting Items for Systematic Reviews and Meta-Analyses)(S1 Table) and MOOSE (Meta-analysis of Observational Studies in Epidemiology) statement. We searched PubMed, Ovid database, China National Knowledge Internet (CNKI), VIP Chinese Science and Technique Journals Database, and Wanfang Database on MRSA (the last search conducted on July 20, 2019) using the following search terms: (“methicillin-resistant *Staphylococcus aureus*” or "MRSA") and ("nasal" or "nasopharyngeal") and "China". The references listed in all included articles were also searched to identify additional relevant articles. The language of publication was in English or Chinese.

### Data extraction

Two authors (Man Wu, Xiang Tong) independently extracted data from all eligible publications. Each study provided the following information: the first author, year of publication, study area/cities, study time, study period, study population, sample size, total numbers of S.*aureus* and MRSA, or resistant of MRSA isolates to commonly available antimicrobial agents, or influencing factors. When the data for analysis was missing in the study, we contacted the author by email. If the author did not respond, the article was excluded. Any disagreement was also resolved through the discussion of the entire group.

### Quality assessments

Two authors independently assessed the quality of included studies using a validated prevalence study quality assessment tool[11], which based on the following eight components: (1) a clear definition of the target population; (2) representative of probability sampling; (3) sample characteristics matching the overall population; (4) adequate response rate (If the sample sociodemographic characteristics match the overall population, the minimum rate should be set at 70%, otherwise 80%); (5) standardized data Collection methods; (6) reliability of survey instruments; (7) validation of survey instruments; and (8) appropriate statistical methods. For "No" and "Yes", the answers were scored 0 or 1. The total quality score for each study varied from 0 to 8. The total scores of 0–4 and 5–8 were considered to be low and non-low quality, respectively. Two authors assessed the quality scores for each study separately and resolved any disagreement through the discussion of the entire group.

### Statistical analysis

All statistical analyses were performed by STATA 12.0. A random effects model (DerSimonian Laird method)[12]was used to obtain a pooled prevalence and a corresponding 95% confidence interval (CI). In a secondary analysis, we also calculated the resistance of MRSA to specific antibiotics. Statistical heterogeneity between groups and within groups was estimated using Chi-square based Q statistic, with *P* values < 0.1 or I^2^ > 50% as statistically significant heterogeneity[13]. Freeman-Tukey double arcsine transformation was used to address both the problem of CIs outside the 0..1 range and that of variance instability[14]. Meta-regression analysis was used to analyze the influencing factors of inter-heterogeneity. We defined logit(*P*) as the dependent variable (*P* referred to the prevalence of MRSA). All the independent factors were selected based on the availability of relevant information in the included studies, including the region (Mainland China, Taiwan, Hong Kong), age range (children, non-children), Study population (Livestock-related workers, children, community residents, healthcare workers, medical students), study period (2001–2010, 2011–2016), and method (*mecA* gene, cefoxitin disk diffusion method, oxacillin disk diffusion method, oxacillin agar dilution method, and others). The factors were included into the random effects meta-regression model with restricted maximum likelihood (REML) method and were analyzed by Odds Ratios (ORs) and 95% CIs. The subgroup analysis was based on the study population, age range, region, study period, and method. A Q-test for heterogeneity was used to compare the effect size in two or more subgroups by assessing the dispersion of the summary effects about the combined effect. Begg's test and Egger's test were used to assess potential publication bias, with *P* < 0.05 indicating potential bias. In addition, sensitivity analysis was used to assess the influence of each study.

## Results

### Characteristics of the studies and assessment of quality

**Fig 1:**
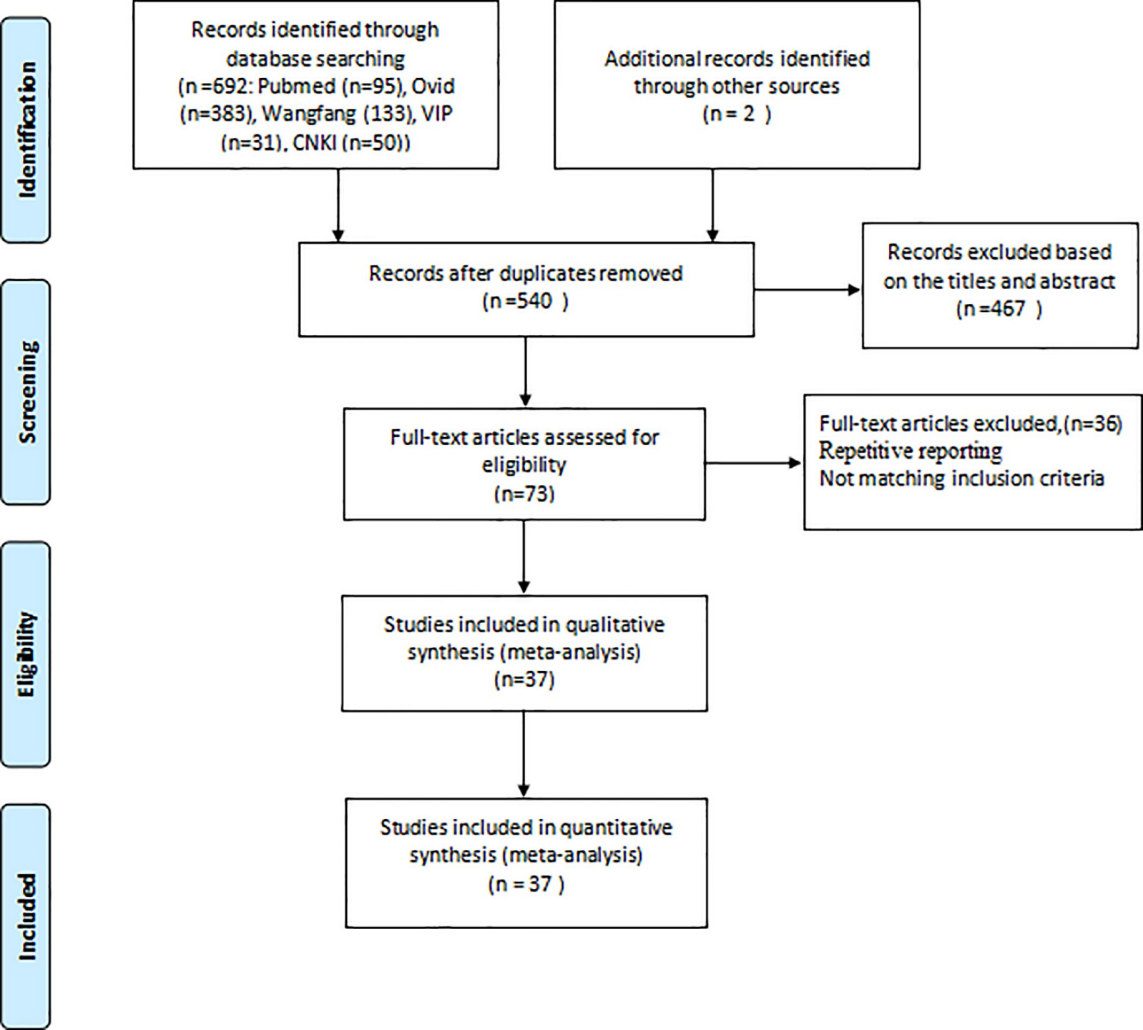
Flow diagram of the selection process.

A total of 694 studies were initially identified from different databases.130 studies were excluded because they were duplicated across the databases. After reading their titles and abstracts, we excluded 467 reviews, meta-analyses, and articles that were not relevant to our study. After the full-text versions were read, we further excluded 36 studies that did not offer usable data (unreported the total number of S.*aureus* or MRSA, repeatability reports). Finally, 37 studies met our inclusion criteria and were included in the meta-analysis [15–50](Fig 1). MRSA identification method differed in the 37 eligible studies, 15 studies were based on the *mecA* gene [15–17, 19–21, 23–25, 28, 29, 31, 35, 41, 44], eight were cefoxitin disk diffusion method [27, 29, 32, 33, 40, 46, 48, 50], three were oxacillin disk diffusion method [7, 39, 42], three were oxacillin agar dilution method [22, 37, 47], only one was ceftizoxime agar plates method[18], and the others were Clinical and Laboratory Standards Institute (CLSI) without specific method. Seven articles were in Chinese and 30 articles were in English. In the 37 eligible studies, 29 studies were cross-sectional studies, six prospective studies only performed one nasal swab and data collection for each participant, additionally, eight nasal swab per participant was performed during one prospective study [49], and nasal samples were obtained during three study periods within 1 year in another prospective study [47]. The main characteristics of the included studies and quality scores were shown in Table 1. The quality score of all cross-sectional studies was 5 to 8 points, with an average of 6.8 points (S2 Table).

**Table 1:** Characteristics of all the included studies.

First author, publication year	Study period	region	Study population	Age range	Sample size, N	No of SA	MRSA prevalence, n(%)	Study type	Score
Ye XH, 2015[15]	2013–11 to 2014–11	Guangdong	Livestock-related workers / Community residents	15-60y	682/1178	91/109	48 (52.7)/16 (14.7)	cross-sectional	8
Fan J, 2011[16]	2005–9 to 2005–9	Sichuan	Healthy children	2-7y	801	147	9 (6.1)	cross-sectional	7
Zhang WJ, 2011[17]	2008–10 to 2009–11	BJ、SH、GZ、JN、YZ	Livestock-related workers	-	51	12	1 (8.3)	cross-sectional	5
Ma XX, 2011[18]	2008–5 to 2009–10	Shenyang	Medical students	19-23y	2103	234	22 (9.4)	cross-sectional	7
Ma XX, 2011[19]	2010	Shenyang	Medical students	21.1y	1634	115	24 (20.9)	cross-sectional	7
Chen B, 2015[20]	2013–10 to 2014–3	Guangzhou	Community residents / Healthcare workers	>17y	297/292	75/63	1 (1.3)/3 (4.8)	cross-sectional	6
Du J, 2011[21]	1 month period	Wenzhou	Medical students	-	935	144	28 (19.4)	cross-sectional	7
O'Donoghue MM, 2004[22]	-	Hong Kong	community residents	11-60y	653	186	9 (4.8)	cross-sectional	6
Xie XY, 2018[23]	2016–2 to 2016–3	Guangzhou	Healthcare workers	20-56y	434	87	10 (11.5)	cross-sectional	8
Yan X, 2015[24]	2009 to 2011	BJ、HRB	Community residents	18-74y	2448	403	8 (2)	cross-sectional	8
Chen BJ, 2017[25]	2014–10 to 2015–5	Guangzhou	Medical students	10-76y	295	73	1 (1.4)	cross-sectional	7
Chen CH, 2018[26]	2005–10 to 2010–12	Taiwan	Healthy children	2-60y	3020	840	246 (29.3)	prospective	8
Deng JJ, 2012[27]	2005 to 2007, 2008 to 2010	Chengdu	Healthy children	2-18y	2373	430	27 (6.3)	cross-sectional	6
Zhang M, 2011[28]	—	Hong Kong	Community residents / Healthcare workers	-	775/249	186/51	4 (2.2)/8 (15.7)	cross-sectional	8
Ho PL, 2012[29]	2009–9 to 2010–4	Hong Kong	Healthy children	2-6y	2211	610	28 (4.6)	cross-sectional	8
Chen CJ, 2011[7]	2005–7 to 2008–6	Taiwan	Healthy children	2-60m	6057	1404	473 (33.7)	cross-sectional	7
Gong ZR, 2017[30]	2012–10 to 2012–11	Tibetan	Healthy children	6-11y	314	16	3 (18.8)	cross-sectional	7
Boost M.V, 2011[31]	-	Hong Kong	Livestock-related workers	20-59y	150	22	2 (9.1)	cross-sectional	7
Fu JJ, 2015[32]	2011–3 to 2011–5	Guangzhou	Healthy children	2.5-12y	1475	550	28 (5.1)	cross-sectional	5
Ge YL, 2012[33]	2009–7 to 2010–6	Shanghai	Healthcare workers	-	2653	152	48 (31.6)	cross-sectional	6
Liu H, 2016[34]	2007 to 2014	Tianjin	Healthcare workers	20-62y	1085	89	12 (13.5)	cross-sectional	6
Zhong JJ, 2016[35]	2013–10 to 2013–12	Guangdong	Livestock-related workers	17-67y	411	51	20 (39.2)	cross-sectional	7
Huang YC, 2007[36]	2005–7 to 2006–10	Taiwan	Healthy children	2m-5y	3046	713	221 (31)	cross-sectional	8
Lu PL, 2005[37]	2001–4 to 2001–10	Taiwan	Community residents / Healthy children / Healthcare workers	1-90y/2-18y/17-60y	851/987/137	149/314/31	31 (20.8)/33 (10.5)/7 (22.6)	cross-sectional	7
Lo WT, 2006[38]	2003–12 to 2005–11	Taiwan	Healthy children	<14y	1195	300	89 (29.7)	prospective	7
Huang YC, 2005[39]	2001–11 to 2002–6	Taiwan	Healthy children / health care workers	-/-	262/137	95/38	5 (5.3)/18 (47.4)	cross-sectional	6
Chen CS, 2012[40]	-	Taiwan	Medical students	18-41y	322	62	7 (11.3)	cross-sectional	6
Wang JT, 2009[41]	2007–10 to 2007–12	Taiwan	Community residents	>18y	3098	686	119 (17.3)	cross-sectional	7
Pan HH, 2017[42]	2005–7 to 2010–12	Taiwan	Healthy children	2-60m	3144	545	165 (30.3)	prospective	7
Wang HK, 2017[43]	2013–6 to 2013–9	Taiwan	Community residents	18-35y	259	58	4 (6.9)	cross-sectional	6
Wu TH, 2018[44]	2015–2 to 2015–6	Taiwan	Healthcare workers	19-91y	326	85	20 (23.5)	prospective	7
Lo WT, 2010[45]	2004 to 2009	Taiwan	Healthy children	1m-14y	3200	824	371 (45)	prospective	7
Huang YC, 2015[46]	2011	Taiwan	Community residents	-	262	73	21 (28.8)	prospective	7
Lu PL 2008[47]	2002–9	Taiwan	Community residents	-	410	112	7 (6.3)	prospective	7
Qu F, 2010[48]	2007–5 to 2007–7	Guangzhou	Community residents	18-31y	1044	209	0 (0)	cross-sectional	7
Chen CJ, 2013[49]	2010–8 to 2011–7	Taiwan	Healthy children	-	154	75	12 (16)	prospective	8
Chang CJ, 2015[50]	2014–6 to 2014–8	Taiwan	Community residents / Healthcare workers	21-77y	75/111	10/17	1 (10)/4 (23.5)	cross-sectional	6

### Meta-analysis results

#### Overall pooled prevalence

**Fig 2:**
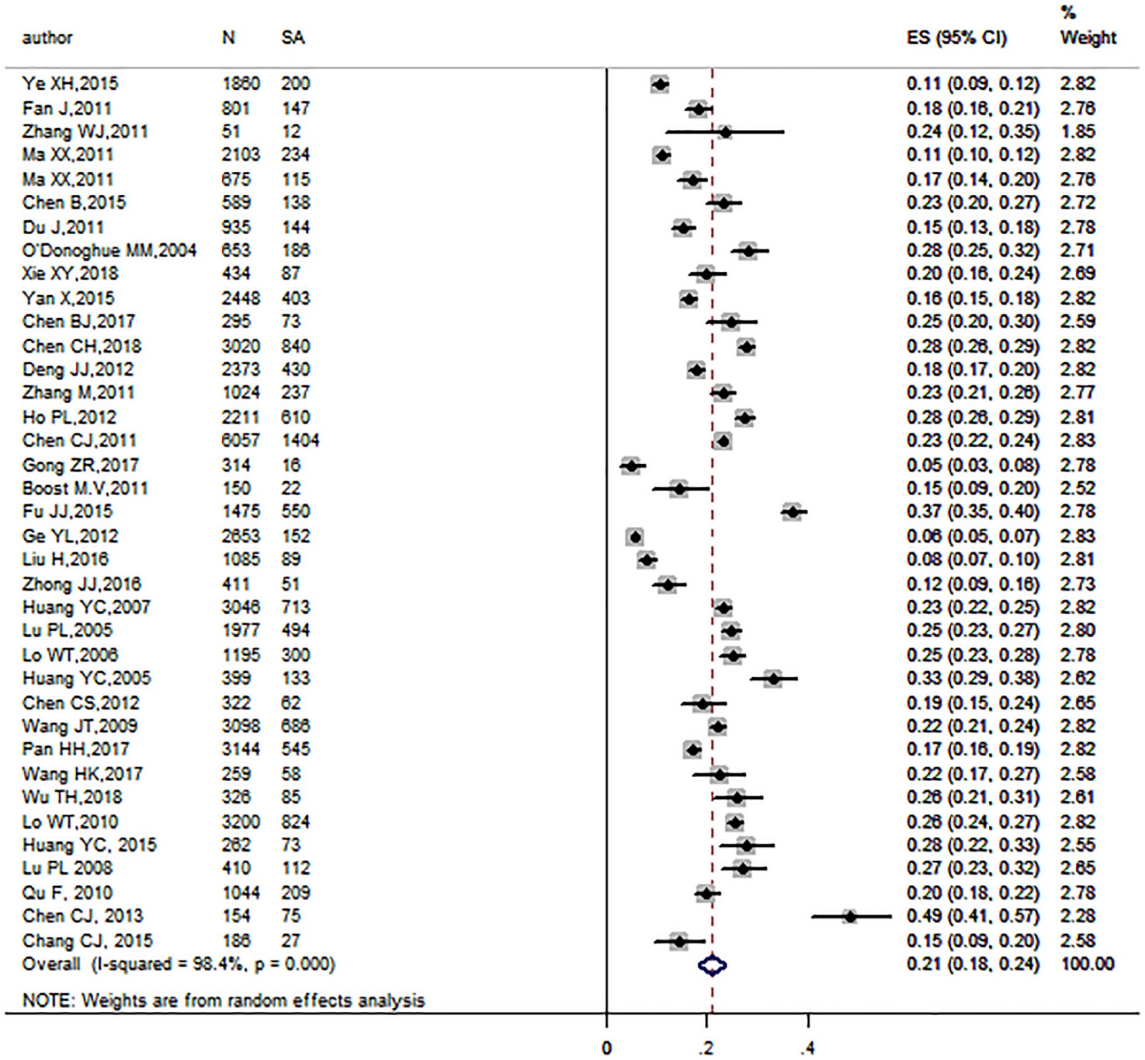
Forest plot for S.*aureus* prevalence and 95% CI for all selected studies.

**Fig 3:**
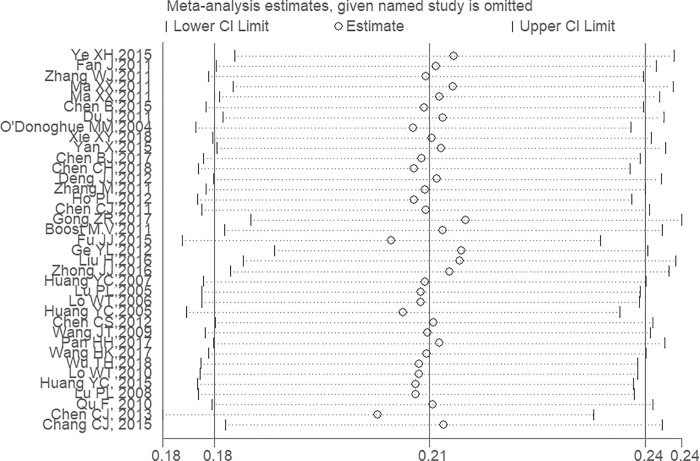
Sensitivity analysis of S.*aureus* prevalence.

In the 37 studies included in the meta-analysis, 10536 S.*aureus* strains were detected from 50639 samples. There was a high level of heterogeneity(I^2^ = 98.4%, *P* < 0.001), therefore, a random effects model was conducted to obtain the pooled prevalence of S.*aureus* among the population (21.2%, 95% CI: 18.5%-23.9%)(Fig 2). Moreover, we performed a sensitivity analysis to explore the effect of every study on the pooled prevalence of S.*aureus*, and no substantial differences were found in the conclusions, indicating the stability of our meta-analysis (Fig 3). No publication bias was detected in Begg’s test(*P* = 0.927) or Egger’s test (*P* = 0.874).

**Fig 4:**
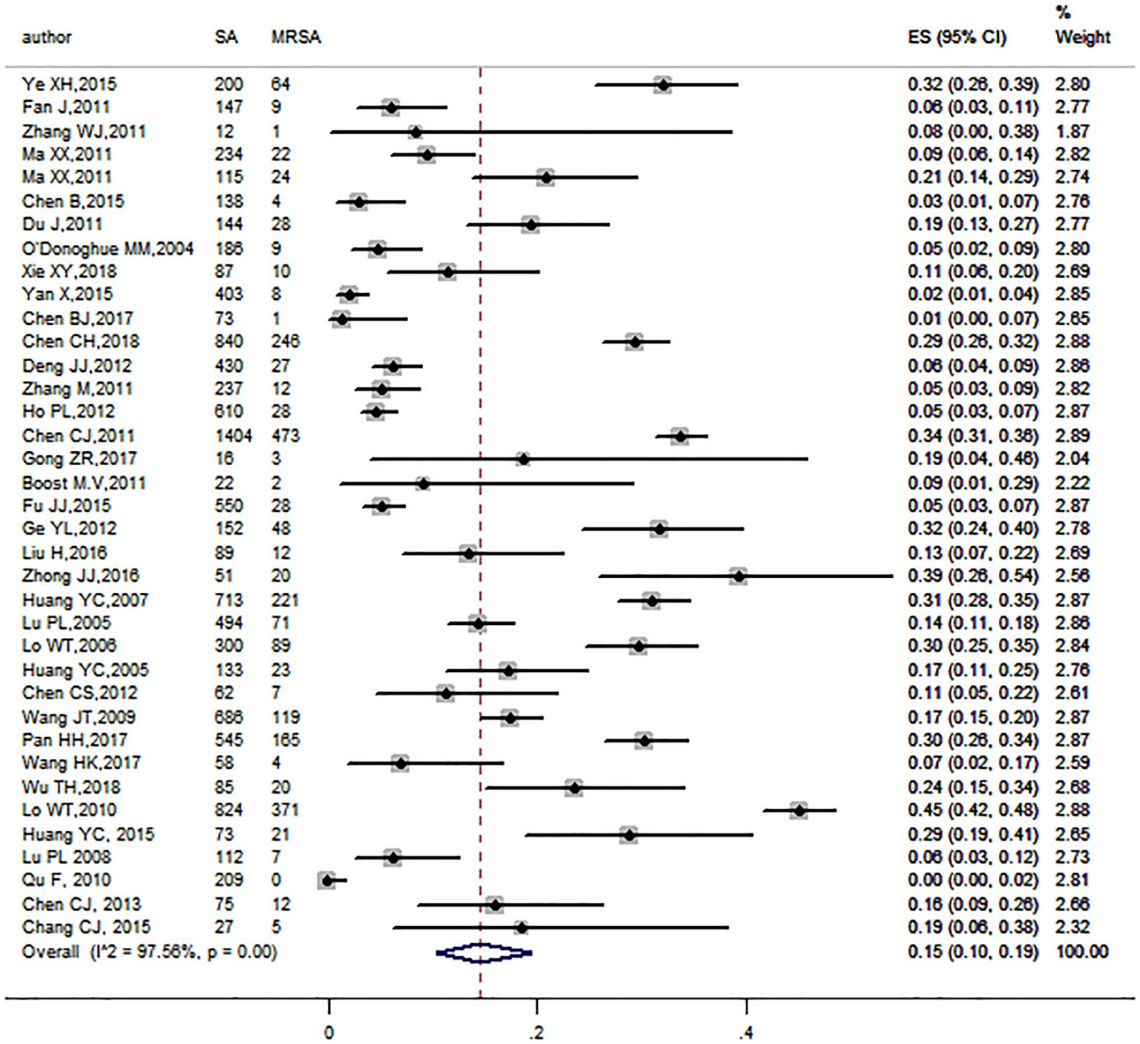
Forest plot of the pooled prevalence of MRSA.

2214 MRSA strains were detected in the included articles, we observed the prevalence of MRSA among S.*aureus* ranging from 0% to 52.7%. A significant heterogeneity was found among the 37 studies (I^2^ = 97.56%, P<0.01), thus, the pooled prevalence of MRSA colonization was 15% (95% CI: 10%-19%)(Fig 4) by a random effect method. No publication bias was detected in Begg’s test (*P* = 0.855).

#### Subgroup analyses

**Fig 5:**
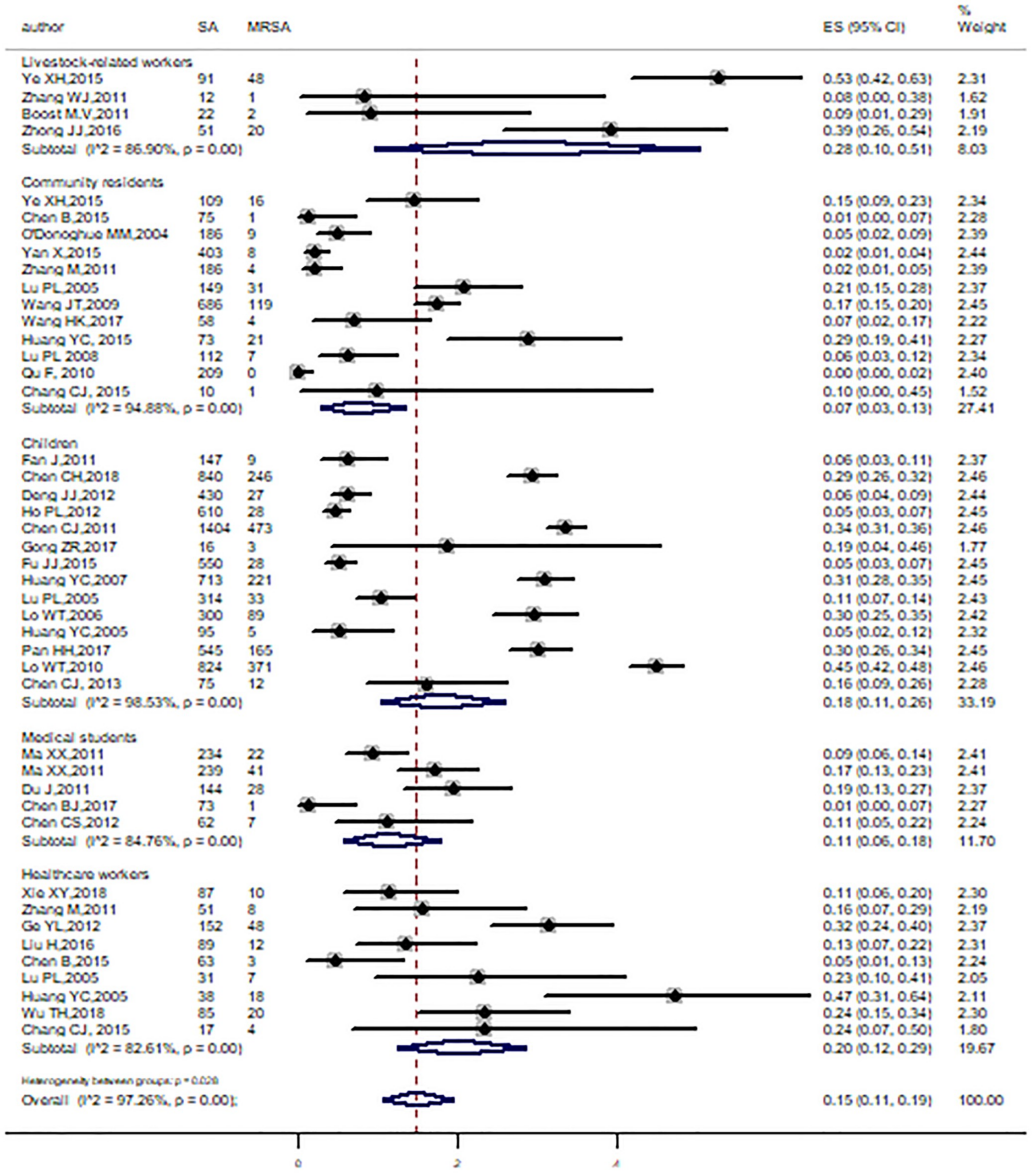
Subgroup analysis for the prevalence of MRSA by population.

**Fig 6:**
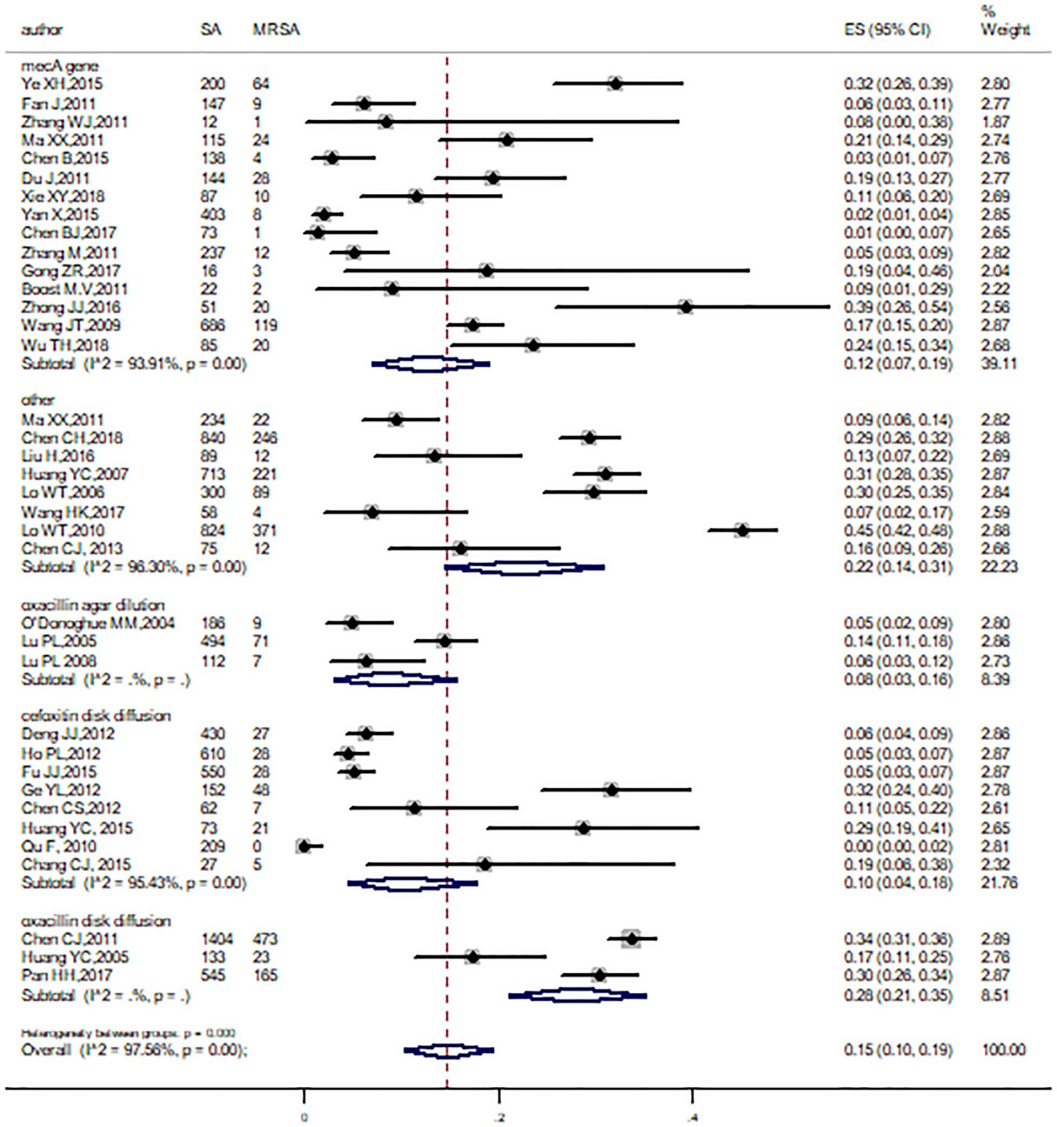
Subgroup analysis for the prevalence of MRSA by methods identification of MRSA.

**Fig 7:**
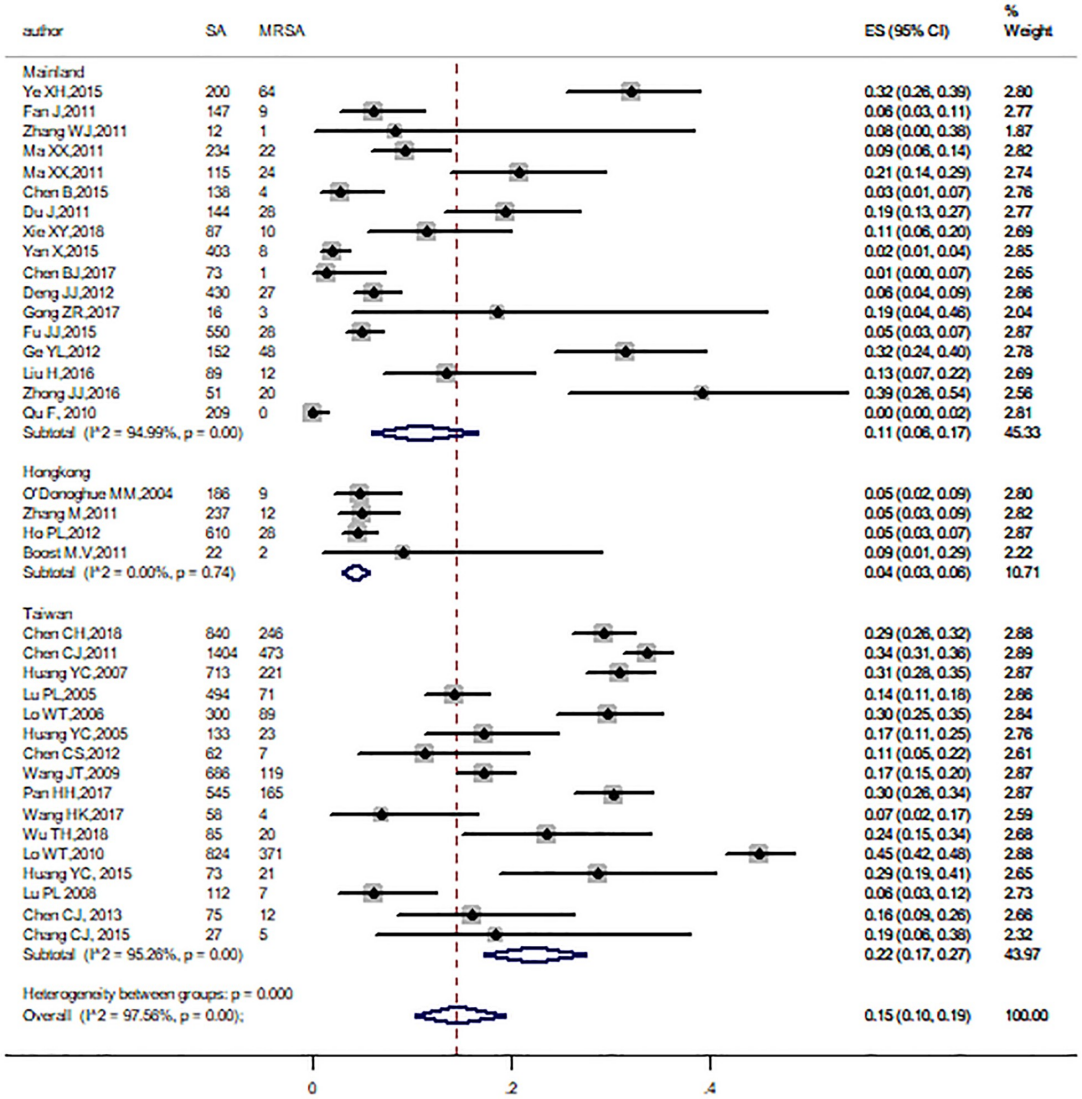
Subgroup analysis for the prevalence of MRSA by region.

Subgroup analyses were conducted by age range, region, study period, method, and study population. All the pooled prevalence of MRSA and corresponding 95% CI of subgroups were obtained, which were showed in Table 2. Among these subgroups, heterogeneity did still exist, except in Hong Kong. Significant differences were also found across study population (P = 0.03), methods (P < 0.001), and regions (P < 0.001). The pooled prevalence of MRSA was 28% (95%CI: 10%-51%, I^2^ = 86.9%) for Livestock-related workers, 18% (95%CI: 11%-26%, I^2^ = 98.53%) for children, 20% (95%CI: 12%-29%, I^2^ = 82.61%) for healthcare workers, 7% (95%CI: 3%-13%, I^2^ = 94.88%) for community residents and 11% (95%CI: 6%-18%, I^2^ = 84.76%) for medical students (Fig 5). In view of different MRSA identification methods, the pooled prevalence in studies was 28% (95%CI: 21%-35%) with oxacillin disk diffusion method, followed by others (22%, 95%CI: 14%-31%, I^2^ = 96.3%), *mecA* gene method (12%, 95%CI: 7%-19%, I^2^ = 93.91%), cefoxitin disk diffusion method (10%, 95%CI: 4%-18%, I^2^ = 95.43%), and the minimum was 8% (95%CI: 3%-16%) with oxacillin agar dilution method (Fig 6). In addition, MRSA proportion was higher in studies conducted in Taiwan than Mainland China and Hong Kong (Fig 7). On the other hand, significant differences were not found across the study period and age ranges (S1 File).

**Table 2:** Pooled prevalence of MRSA among S.*aureus* estimates by subgroups.

Subgroups	Number of studies	MRSA prevalence(%)	95%Confidence Interval	I^2^(*P*-value)	*P*-value ^a^
**Age range**					
Children	14	18	11–26	98.53 (P<0.001)	0.4
Non-children	30	13	9–18	93.7 (P<0.001)	
**Region**					
Mainland China	17	11	6–17	94.99 (P<0.001)	<0.001
Taiwan	16	22	17–27	95.26 (P<0.001)	
Hong Kong	4	4	3–6	0 (P = 0.74)	
**Study population**					
Community residents	12	7	3–13	94.88 (P<0.001)	0.03
Livestock-related workers	4	28	10–51	86.9 (P<0.001)	
Children	14	18	11–26	98.53 (P<0.001)	
Healthcare workers	9	20	12–29	82.61 (P<0.001)	
Medical students	5	11	6–18	84.76(P<0.001)	
**Study period**					
2001–2010	19	16	11–23	98.2 (P<0.001)	0.43
2011–2016	18	13	8–19	92.77 (P<0.001)	
**Method**					
*mecA* gene	15	12	7–19	93.91 (P<0.001)	<0.001
cefoxitin disk diffusion	8	10	4–18	95.43 (P<0.001)	
oxacillin disk diffusion	3	28	21–35	-	
others	8	22	14–31	96.3 (P<0.001)	
oxacillin agar dilution	3	8	3–16	-	

^a^ a Q-test for heterogeneity between subgroups

#### Meta-regression for the prevalence of MRSA

The meta-regression was performed to identify related potential influencing factors of inter-heterogeneity, which was showed in Table 3. The meta-regression (residual I^2^ = 99.49%, adj R^2^ = 12.15%, P = 0.028 in the test for the goodness of model fit) showed that compared with Taiwan, the prevalence of MRSA was significantly lower in Mainland China (OR = 0.43, 95%CI: 0.2–0.92, P = 0.003), and Hong Kong (OR = 0.26, 95%CI: 0.08–0.84, P = 0.026). In addition, the prevalence of MRSA was higher among Livestock-related workers (OR = 4.54, 95%CI: 1.23–16.77, P = 0.024), children (OR = 2.85, 95%CI: 1.19–6.83, P = 0.02) and healthcare workers(OR = 3.67, 95%CI: 1.31–9.79, P = 0.011) than community residents.

**Table 3:** Summary results of meta-regression for the prevalence of MRSA.

Factor	Coefficient	OR	95% CI (OR)	P-value
**Age range**				
Non-children	-	1	-	-
children	0.405	1.499	0.623–3.301	0.307
**Region**				
Taiwan	-	1	-	-
Mainland	-0.84	0.432	0.203–0.919	0.003
Hong Kong	-1.359	0.257	0.078–0.842	0.026
**Study population**				
Community residents	-	1	-	-
Children	1.048	2.851	1.191–6.825	0.02
Livestock-related workers	1.512	4.537	1.228–16.769	0.024
Medical students	0.417	1.517	0.415–5.544	0.519
Healthcare workers	1.296	3.655	1.314–9.794	0.011
**Study period**				
2001–2010	-	1	-	-
2011–2018	-0.314	0.73	0.349–1.53	0.396
method				
*mecA* gene	-	1	-	-
cefoxitin disk diffusion	-0.381	0.683	0.253–1.844	0.442
oxacillin disk diffusion	1.117	3.054	0.939–9.936	0.063
others	0.706	2.026	0.82–5.005	0.122

#### Influencing factors

Among the 37 articles, only seven studies reported risk factors for MRSA carriage among healthy Chinese population. The significant risk factors were identified through univariable or multivariable logistic regression models, and they included younger age (OR: 3.54, 95% CI: 2.38–5.26; OR: 2.24, 95% CI: 1.73–2.9), attending day care centers (DCCs) (OR: 1.95, 95% CI: 1.4–2.72; OR: 1.53, 95% CI: 1.2–1.95), flu vaccination (OR: 1.73, 95% CI: 1.28–2.35), residing in northern Taiwan (OR: 1.45, 95% CI: 1.19–1.77) in children, contact with livestock (OR: 6.31, 95% CI: 3.44–11.57) in Livestock-related workers, regular visits to health care facility (OR: 23.83, 95% CI: 2.72–209.01), household member working in health care facility (OR: 8.98, 95% CI:1.4–55.63), and using antibiotics within the past year (OR: 2.05, 95% CI:1.35–3.11). While colonization by S.pneumoniae (OR: 0.7, 95% CI: 0.52–0.94), Smoking habits (OR:0.44, 95% CI: 0.24–0.82) and breastfeeding (OR: 0.69, 95% CI: 0.516–0.93; OR: 0.65, 95% CI: 0.53–0.8) were protective factors in against MRSA carriage. Other influencing factors were reported in Table 4.

**Table 4:** Risk factors of MRSA nasal carriage in healthy Chinese population reported in the selected studies.

Influencing Factors, Odds ratio (95% CI)	Univariable logistic regression models				Multivariable logistic regression models		
	Xie XY, 2018[23]	Chen CH, 2018 [26]	Chen CJ, 2011 [7]	Chen CJ, 2013[49]	Ye XH, 2015[15]	Wang JT, 2009 [41]	Pan HH, 2017 [42]
Gender (male vs female)	0.88 (0.24–3.16)	-	-	-	-	-	0.99 (0.72–1.36)
Age (2–6 m vs 0.5-5y)	-	3.54 (2.38–5.26) *	2.24 (1.73–2.9)*	-	-	-	-
Current smoking statue (yes vs no)	2.03 (0.25–16.75)	-	-	-	-	-	-
Residing in northern Taiwan (yes vs no)	-	-	1.45 (1.19–1.77)*	-	-	-	-
Department (microbiological laboratory vs other laboratory)	0.58 (0.12–2.76)	-	-	-	-	-	-
Nasal cleaning habit (daily or weekly vs rarely or never)	1.34 (0.34–5.26)	-	-	-	-	-	-
Underlying disease (yes vs no)	1.88 (0.39–9.2)	-	-	-	-	-	-
Colonization by S. pneumoniae (yes vs no)	-	-	0.7 (0.52–0.94)*	-	-	-	-
Breastfeeding (yes vs no)	-	0.69 (0.52–0.93)*	0.65 (0.53–0.8)*	-	-	-	0.85 (0.60–1.20)
Day care attendance (yes vs no)	-	1.95 (1.4–2.72)*	1.53 (1.2–1.95)*	-	-	-	0.78(0.48–1.24)
Flu vaccination (yes vs no)	-	1.73 (1.28–2.35)*	-	-	-	-	-
Contact with livestock (yes vs no)	-	-	-	-	6.31 (3.44–11.57)*	-	-
Contact with pig(yes vs no)	-	-	-	-	6.58 (3.50–12.38)*	-	-
Contact with poultry(yes vs no)	-	-	-	-	4.94 (1.32–18.41)*	-	-
Contact with other animal(yes vs no)	-	-	-	-	4.50 (0.88–22.98)*	-	-
Living with hospital staff (yes vs no)	0.83(0.21–3.27)	-	-	-	-	-	-
Smoking habits(yes vs no)	-	-	-	-	-	0.44(0.24–0.82)*	-
Passive smoking (yes vs no)	-	-	-	-	-	-	1.16 (0.84–1.60)
Using antibiotics within the past year (yes vs no)	-	-	-	**-**	-	**2**.05 (1.35–3.11)*	**-**
Presence of household members aged under 7	-	-	-	-	-	2.24 (1.53–3.29)*	-
Received antibiotics within 2 weeks (yes vs no)	-	-	-	-	-	-	1.78 (0.92–3.46)
URI within 2 weeks (yes vs no)	-	-	-	-	-	-	1.270(0.88–1.84)
Pneumococcal vaccination(yes vs no)	-	-	-	-	-	-	1.38(0.89–2.14)
Regular visits to health care facility(yes vs no)	-	-	-	23.83(2.72–209.01)*	-	-	-
Household member working in health care facility(yes vs no)	-	-	-	8.98 (1.4–55.63)*	-	-	-

*Statistical significance

Underlying disease: hypertension, diabetes, chronic rhinitis, urticaria, hyperthyroidism

Furthermore, 10 studies were included for meta-analysis of the antimicrobial resistance of MRSA isolates. The pooled prevalence of MRSA resistance for 11 antibiotics included in the meta-analysis were presented in Table 5. High prevalence of resistance was observed to penicillin (100%, 95% CI: 99%-100%, P<0.001), erythromycin(88%, 95% CI: 79%-95%, P<0.001), Clindamycin (75%, 95% CI: 60%-87%, P<0.001). However, linezolid had a low rate of resistance (0%, 95% CI: 0%-4%, P<0.001), and the resistance of MRSA to Vancomycin has not been found in healthy people.

**Table 5:** Pooled prevalence of MRSA resistance to different antimicrobial agents in healthy Chinese population.

Antibiotics R (%)	Fan J, 2011	Chen B, 2015	Du J, 2011	Fu JJ, 2015	Zhong JJ, 2016	Huang YC, 2007	Huang YC, 2005	O'Donoghue MM, 2004	Chen CS, 2012	Pan HH, 2017	Pooled resistance rate, (95% CI)	I^2^ (*P*-value)
Penicillin	9 (100)	-	28 (100)	25 (89.3)	20 (100)	210 (99.1)	23 (100)	9 (100)	-	162 (98.2)	1 (0.99–1)	0 (*P* = 0.48)
Ampicillin	-	-	26 (92.9)	-	-	-	-	-	-	-	0.93 (-)	-
Erythromycin	7 (77.8)	4 (100)	21 (75)	23 (82.1)	12 (60)	198 (93.4)	23 (100)	7 (77.8)	5 (71.4)	153 (92.7)	0.88 (0.79–0.95)	71.89 (*P*<0.001)
Gentamicin	-	1 (25)	8 (28.6)	-	2 (10)	-	-	5 (55.6)	-	-	0.26 (0.09–0.47)	53.13 (*P* = 0.09)
Clindamycin	2 (22.2)	4 (100)	15 (53.6)	22 (78.6)	13 (65)	193 (91)	-	7 (77.8)	5 (71.4)	147 (89.1)	0.75 (0.6–0.87)	84.23 (*P*<0.001)
Tetracycline	-	1 (25)	8 (28.6)	8 (28.6)	10 (50)	-	-	-	-	-	0.33 (0.22–0.44)	0 *(P* = 0.42)
Ciprofloxacin	-	-	15 (53.6)	-	3 (15)	-	-	5 (55.6)	-	-	0.39 (0.13–0.69)	77.04 *(P* = 0.01)
Levofloxacin	-	-	11 (39.3)	1 (3.6)	-	-	-	-	-	-	0.18 (0.09–0.3)	-
Doxycycline	-	-	-	-	-	5 (2.4)	-	-	0 (0)	8 (4.9)	0.02 (0–0.04)	0 (P = o.43)
Vancomycin	0 (0)	-	0 (0)	0 (0)	-	0 (0)	0 (0)	-	0 (0)	0 (0)	0 (0–0)	0 (*P* = 0.93)
Linezolid	1 (11.1)	-	0 (0)	0 (0)	1 (5)	-	-	-	0 (0)	-	0 (0–0.04)	0 (*P* = 0.41)

## Discussion

Nasal MRSA carriage has been extensively studied in a variety of study populations with significant heterogeneous prevalence and influencing factors. We conducted this meta-analysis to summarize the prevalence of MRSA, antibiotic resistance and influencing factors of MRSA carriage in healthy Chinese population. The main findings were as follows: the pooled prevalence of MRSA was about 21%, and the prevalence of S.aureus was about 15%. When performing a subgroup analysis by study population, children (18%), Livestock-related workers (28%) and healthcare workers (20%) presented higher prevalence of MRSA compared with community residents (7%). When classifying studies by region, the prevalence of MRSA carriage in Taiwan (22%) was higher than in mainland China (11%) and Hong Kong (4%). The risk factors of MRSA carriage were including living in northern Taiwan, younger age, attending DCCs, flu vaccination, using antibiotics within the past year, working in hospital, and contact Livestock or medical environment. These MRSA strains also showed extreme resistance to penicillin, ampicillin, erythromycin and clindamycin (100%, 93%, 88%, and 75%).

The prevalence of S.*aureus* was lower in our meta-analysis compared to diabetes population that was investigated by Lin J *et al* in the US (21.2% vs 28.3%)[51]. The prevalence of MRSA among S.*aureus* was about 15%, which was far lower than the average prevalence of clinical isolate MRSA reported by CHINET surveillance of bacterial resistance across China (38.4% between January 1, 2016 to December 31, 2016)[52]. However, Den Heijer CD *et al* conducted a study to find that the highest prevalence of MRSA was 2.1% and prevalence of S.*aureus* was 21.6% (ranging from 12.1% to 29.4%) for healthy people in nine European countries[53]. The lower prevalence of MRSA in European countries may be the result of increased public awareness of MRSA and subsequent public health measures to control MRSA.

As showed in subgroup analysis by region, the prevalence of MRSA in Taiwan was 22%, which was higher than the mainland and Hong Kong (11%, 4%). Meta-regression suggested that Taiwan was an risk factor of MRSA nasal carriage, which may be due to genetic variability or infection control measures. Further research is needed. In addition, the highest prevalence of MRSA was observed in the Livestock-related workers, followed by healthcare workers, children, medical students, community residents(28%, 20%, 18%, 11%, 7%). In this meta-regression, contact animals, children and working in hospital were risk factors for MRSA carriage. Several recent studies have shown that occupational livestock contact might lead to livestock-associated methicillin-resistant *Staphylococcus aureus* (LA-MRSA) transmission to humans, and LA-MRSA strains were associated with severe and lethal infections in humans[15, 54, 55]. Therefore, the emergence of LA-MRSA may pose a potential public health hazard that requires continuously monitoring. In addition, a review conducted by Dulon M *et al* found that carriage prevalence among healthcare works are much higher than among community members in Europe and the United States[56]. Not surprisingly, we also found Healthcare workers was the risk of MRSA carriage, which may be explained by the frequent and intimate contact with patient in the medical environment. Children were also considered to be a risk factor of MRSA in our meta-analysis, and may be a reservoirs of MRSA and play an important role in MRSA dissemination[57]. As for subgroup of methods to identify MRSA, The prevalence of MRSA in studies with oxacillin disk diffusion method (28%) seemed higher than that with the mecA gene method(12%), however, there was not statistically significant by meta-regression analysis. It is well known that the mecA gene method is recognized as the gold standard for diagnosing MRSA, which is generally only suitable to identification of purified *staphylococcus* cultures[58]. However, conventional antibiotic susceptibility tests, such as cefoxitin and oxacillin disc diffusion, have become the mainstream of diagnostic MRSA[59].

Moreover, seven articles discussed the influencing factors for MRSA nasal carriage in healthy Chinese populations. Younger age, attending DCCs, flu vaccination, living in northern Taiwan, using antibiotics within the past year, and frequent contact with livestock and medical environment are independent predictors of MRSA carriage. Among environmental factors, crowded environments, such as attending DCCs and living in northern Taiwan with the smaller mean house size compared with southern Taiwan, were associated risk for subsequent MRSA colonization. Antibiotic usage was an independent risk of MRSA colonization, so, health care providers should promote the rational use of antibiotic. One of the seven studies unexpectedly found that influenza vaccination were significantly associated with MRSA colonization without confirmed by any research[26]. However, colonization by S. *pneumoniae* and breastfeeding are protective factors. Host innate immunity is associated with S.*aureus* nasal colonization, and breastfeeding may play a protective role in MRSA colonization through immunity[7, 60]. Additionally, the relationship between the prevalence of MRSA carriage and colonization by S. *pneumoniae* may be elucidated by S. *pneumoniae*-S. *aureus* interference, which could be mediated by hydrogen peroxide in the vitro study conducted by Gili RY *et al*[61]. Smoking habits appeared to be a protective factor of MRSA carriage in our study, while parental smoking were independent risk factors in children[62]. Clearly, the impact of smoking on MRSA colonization needs further research.

Based on the above discussion, public health departments should focus on the results of meta-regression analysis and significant influencing factors when establishing public health interventions to reduce MRSA infection. Public health departments should pay more attention to healthy population in china with younger age, attending DCCs, flu vaccination, using antibiotics within the past year, working in hospital, and contact Livestock or medical environment.

In this meta-analysis, we also estimated the pooled prevalence of MRSA resistance to 11 different antimicrobial agents commonly used in China. It was found that MRSA resistance to commonly available antimicrobial agents in China was ranging from 0% to vancomycin to 100% to penicillin. MRSA resistance to beta-lactam antibiotics (penicillin, ampicillin) is understood via expressing PBP2a[63]. In addition, MRSA was highly resistant to erythromycin and clindamycin (88%, 75%). Several previous studies have found that MRSA resistance to erythromycin is also associated with resistant to clindamycin, this cross-resistance can be mediated by erythromycin ribosomal methylase encoding genes[64, 65]. In 2002, the first vancomycin-resistant S. *aureus* strain was reported in the United States[66]. Fortunately, no resistance to vancomycin was found in our meta-analysis. However, more and more vancomycin intermediate-resistant S. *aureus* (VISA) was reported with increasing frequency in the use of vancomycin, which may pose severe challenges to public health security in the future[67]. At present, vancomycin remains the first choice for the treatment of serious MRSA infection. In addition, Linezolid and daptomycin are considered as the first-line drugs for some selected patients, such as skin and skin structure infections[68].

There are some strengths. Firstly, the included studies have provided sufficient simple size. Secondly, all subjects were healthy Chinese people, thus excluding the impact of ethnic, which was considered as a major potential confounding factor. Most importantly, MRSA poses a serious threat to public health. MRSA colonization in healthy hosts is a risk factor in causing infection and may play an important role in the dissemination of MRSA in community and hospital settings. This meta-analysis is the first to focus on the prevalence of MRSA in healthy Chinese populations, and could provide some epidemiological information about MRSA and the influencing factors, as well as antibiotic resistance.

However, there are also some limits in our meta-analysis. Firstly, the significant heterogeneity of prevalence of MRSA in the included studies was observed. There was no doubt that we should pay attention to heterogeneity. However, our subgroup analysis by age, study population, region, and method did not significantly reduce heterogeneity, except in Hong Kong. Because sufficient data in primary studies were lacking, we failed to perform further subgroup analyses to investigate the other factors, such as gender, history of previous antibiotic usage, smoking, which may also be the cause of such heterogeneity. Secondly, we only focus on nasopharyngeal colonization, however, Bitterman Y *et al* indicated that multiple sites should be used to detect carry-over status[69]. In addition, the included studies are cross-sectional studies, which make it difficult to distinguish between persistent carriers and intermittent carriers[70]. Finally, all the studies are mainly carried out in high-level general hospitals, and the study areas are unevenly distributed in the mainland, mainly in Beijing, Shanghai, Guangzhou, Sichuan, Shenyang and Jinan. Therefore, it is impossible to represent population distribution of the whole country. Despite the above weaknesses of the study, all studies were of non-low quality (score > 5). Additionally, publication bias did not found according to the Begg's test and Egger's test. So the results of this study are reliability and accuracy. In the future, prospective studies may need to verify these results, to guide the development of measures to control the spread of MRSA.

## Conclusion

In this meta-analysis, the pooled prevalence of S.*aureus* was about 21%, and the pooled MRSA prevalence was considerably high, reaching 15%. MRSA was also found to be highly resistant to penicillin, ampicillin, erythromycin and clindamycin. In contrast, MRSA was not found to be resistant to vancomycin in healthy Chinese population. In addition, to control MRSA carriage and infection, Public MRSA protection measures should be required in Livestock-related workers and children with younger age or attending DCCs. Healthcare workers should take strict disinfection measures, and strengthen the surveillance of MRSA. Additionally, the supervision of antibiotics also should be strengthened in both hospitals and communities.

## Supporting information

S1 File Subgroup analyses. (DOCX)

S1 Table PRISMA checklist. (DOC)

S2 Table Quality assessment of the included studies. (DOCX)
