# Supplementary material for: Prevalence of methicillin-resistant Staphylococcus aureus in healthy Chinese population: A system review and meta-analysis
Source: PLoS One. 2019 Oct 24;14(10):e0223599. doi: 10.1371/journal.pone.0223599 (PMC6812772; doi:10.1371/journal.pone.0223599)
Supplement: S1 File — (DOCX) [file pone.0223599.s001.docx]

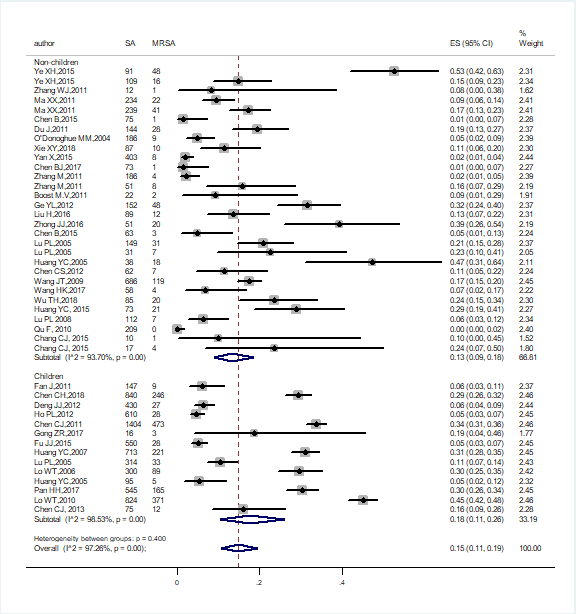


**Fig 8.** **Subgroup analysis for the prevalence of MRSA by age.**


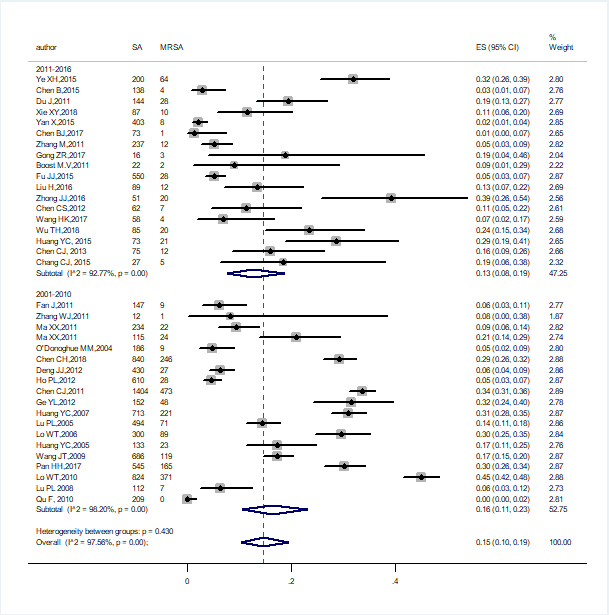


**Fig 9. Subgroup analysis for the prevalence of MRSA by study period.**
