# Supplementary material for: Prevalence of methicillin-resistant Staphylococcus aureus in healthy Chinese population: A system review and meta-analysis
Source: PLoS One. 2019 Oct 24;14(10):e0223599. doi: 10.1371/journal.pone.0223599 (PMC6812772; doi:10.1371/journal.pone.0223599)
Supplement: S2 Table — (DOCX) [file pone.0223599.s003.docx]

| Study ID | Clear definition of the target population | Representative of probability sampling | Sample characteristics matching the overall population | Adequate response rate | Standardised data collection methods | reliability of survey instruments | validation of survey instruments | appropriate statistical methods | Total scores |
| --- | --- | --- | --- | --- | --- | --- | --- | --- | --- |
| Ye XH，2015 | Yes | Yes | Yes | Yes | Yes | Yes | Yes | Yes | 8 |
| Fan J，2011 | Yes | Yes | Yes | Yes | Yes | Yes | Yes | No | 7 |
| Zhang WJ，2011 | Yes | No | No | Yes | Yes | Yes | Yes | No | 5 |
| Ma XX，2011 | Yes | Yes | Yes | Yes | Yes | Yes | No | Yes | 7 |
| Ma XX，2011 | Yes | Yes | Yes | Yes | Yes | Yes | Yes | No | 7 |
| Chen B，2015 | Yes | Yes | NO | NO | Yes | Yes | Yes | Yes | 6 |
| Du J，2011 | Yes | Yes | Yes | Yes | Yes | Yes | Yes | No | 7 |
| O'Donoghue MM，2004 | Yes | No | No | Yes | Yes | Yes | Yes | Yes | 6 |
| Xie XY，2018 | Yes | Yes | Yes | Yes | Yes | Yes | Yes | Yes | 8 |
| Yan X，2015 | Yes | Yes | Yes | Yes | Yes | Yes | Yes | Yes | 8 |
| Chen BJ，2017 | Yes | No | Yes | Yes | Yes | Yes | Yes | Yes | 7 |
| Chen CH，2018 | Yes | Yes | Yes | Yes | Yes | Yes | Yes | Yes | 8 |
| Deng JJ，2012 | Yes | No | Yes | Yes | Yes | Yes | Yes | No | 6 |
| Zhang M，2011 | Yes | Yes | Yes | Yes | Yes | Yes | Yes | Yes | 8 |
| Ho PL，2012 | Yes | Yes | Yes | Yes | Yes | Yes | Yes | Yes | 8 |
| Chen CJ，2011 | Yes | No | Yes | Yes | Yes | Yes | Yes | Yes | 7 |
| Gong ZR，2017 | Yes | Yes | Yes | Yes | Yes | Yes | Yes | No | 7 |
| Boost M.V，2011 | Yes | Yes | Yes | Yes | Yes | Yes | Yes | No | 7 |
| Fu JJ，2015 | Yes | No | No | Yes | Yes | Yes | Yes | No | 5 |
| Ge YL，2012 | Yes | Yes | No | Yes | Yes | Yes | Yes | No | 6 |
| Liu H，2016 | Yes | Yes | Yes | Yes | Yes | Yes | No | No | 6 |
| Zhong JJ，2016 | Yes | Yes | Yes | Yes | Yes | Yes | Yes | No | 7 |
| Huang YC，2007 | Yes | Yes | Yes | Yes | Yes | Yes | Yes | Yes | 8 |
| Lu PL，2005 | Yes | Yes | Yes | No | Yes | Yes | Yes | Yes | 7 |
| Lo WT, 2006 | Yes | No | Yes | Yes | Yes | Yes | Yes | Yes | 7 |
| Huang YC, 2005 | Yes | No | Yes | Yes | Yes | Yes | Yes | No | 6 |
| Chen CS, 2012 | Yes | No | Yes | No | Yes | Yes | Yes | Yes | 6 |
| Wang JT, 2009 | Yes | Yes | Yes | Yes | Yes | Yes | Yes | No | 7 |
| Pan HH, 2017 | Yes | No | Yes | Yes | Yes | Yes | Yes | Yes | 7 |
| Wang HK, 2017 | Yes | No | No | Yes | Yes | Yes | Yes | Yes | 6 |
| Wu TH, 2018 | Yes | No | Yes | Yes | Yes | Yes | Yes | Yes | 7 |
| Lo WT, 2010 | Yes | No | Yes | Yes | Yes | Yes | Yes | Yes | 7 |
| Huang YC, 2015 | Yes | Yes | No | Yes | Yes | Yes | Yes | Yes | 7 |
| Lu PL 2008 | Yes | No | Yes | Yes | Yes | Yes | Yes | Yes | 7 |
| Qu F, 2010 | Yes | No | Yes | Yes | Yes | Yes | Yes | Yes | 7 |
| Chen CJ, 2013 | Yes | Yes | Yes | Yes | Yes | Yes | Yes | Yes | 8 |
| Chang CJ, 2015 | Yes | No | Yes | No | Yes | Yes | Yes | Yes | 6 |

**Table S2. Quality assessment of included studies**
